# The Influence of pH on Complexation Process of Copper(II) Phosphoethanolamine to Pyrimidine Nucleosides

**DOI:** 10.3390/ma14154309

**Published:** 2021-08-01

**Authors:** Malwina Gabryel-Skrodzka, Martyna Nowak, Klaudia Stachowiak, Michal Zabiszak, Kazuma Ogawa, Renata Jastrzab

**Affiliations:** 1 Faculty of Chemistry, Adam Mickiewicz University in Poznan, Uniwersytetu Poznanskiego 8, 61-614 Poznan, Poland; malwina.gabryel@amu.edu.pl (M.G.-S.); martynan@amu.edu.pl (M.N.); klasta5@st.amu.edu.pl (K.S.); zabiszakm@amu.edu.pl (M.Z.); 2 Pharmaceutical Sciences Department, Kanazawa University, Kakuma-machi, Kanazawa 920-1192, Japan; kogawa@p.kanazawa-u.ac.jp

**Keywords:** phosphoethanolamine, pyrimidine nucleosides, copper(II) ion complexes, potentiometric measurements, spectroscopic studies

## Abstract

The influence of pH on the complex formation of phosphoethanolamine and pyrimidine nucleosides (uridine, cytidine and thymidine) with copper(II) ions was studied. All investigations were performed in aqueous solution. The overall stability constants of the complexes and non-covalent compounds were obtained using the potentiometric method with computer calculation of the data. Moreover, equilibrium constants of the reaction were determined. The mode of coordination was obtained using spectroscopic methods. Analysis of the potentiometric and spectroscopic data confirmed the involvement and effectiveness of phosphate groups in species formation as well as the influence of pH on the mode of coordination of the investigated biomaterials. In the next step, studied complexes will be applied as potential biomaterials with biological applications.

## 1. Introduction

The phosphate group that is found in genetic material such DNA and RNA, which activates proteins and is a common buffer in cells, also takes part in keeping acid–base equilibrium [1,2]. Phosphorylation and dephosphorylation are the most important processes of protein modification and signal transmission. The most frequent phosphorylated ligands in living organisms are nucleotides. For pyrimidine nucleosides, the potential non-covalent interactions and metal ion bonding is the donor nitrogen atoms N(3), and also phosphate groups for nucleotides. Other compounds found in organisms containing a phosphate group are phosphoserine, phosphothreonine, phosphocholine and phosphoethanolamine.

Phosphoethanolamine is the intermediate in the synthesis of phosphatidylethanolamine (PE)—one of the most abundant phospholipids in animal and plant lipids [3,4,5]. In most eukaryotic membranes, phosphatidylcholine and phosphatidylethanolamine represent together around 60%–85% of the phospholipid fraction [6]. Phospholipids like PE play an important role in living organisms because they are structural and functional components of biomembranes and play a dynamic role in regulatory processes [7,8,9,10,11].

A significant increase in the cellular concentration of phosphoethanolamine to a level that inhibits mitochondrial respiration results from the action of meclizine (first-generation antihistamine drug). It was proved that the effect of meclizine on cellular respiration was enhanced by ethanolamine. This, in turn, makes meclizine as well as phosphoethanolamine useful in the treatment of infectious diseases (such as malaria or African coma) as well as cancer [12].

One of the microelements that occur in trace amount in living organisms are copper ions. Copper(II) proteins have diverse roles in biological electron transfer and oxygen transportation. Copper(II) ions are essential in the aerobic respiration of all eukaryotes—it is found in cytochrome c oxidase in mitochondria. Copper(II) ions are also found in many enzymes responsible for antioxidation processes (superoxide dismutases) and play a role in processes that work against heart diseases including atherosclerosis [13,14]. The majority of Cu(II) ions in ceruloplasma are in the form of mixed complexes with ligands, such as amino acids, peptides and phosphorylated amies [15,16,17]. Copper(II) complexes have been found to be active both in vitro and in vivo [18] and are reported to act as a potential anticancer and cancer-inhibiting agent [19,20,21]. Understanding the phenomenon of complexation of biomolecules is crucial to clarifying the role of metal ions in living organisms [22,23].

This article presents the results of potentiometric, spectral and theoretical studies of the complexation of phosphoethanolamine with copper(II) in binary and also in ternary systems with pyrimidine nucleosides: cytidine (Cyd), thymidine (Thd) and uridine (Urd). The determination of the stability constants of the complexes, as well as the identification of the mode of coordination, in the systems containing phosphorylated derivative compounds is the first step for explanation of the function of phosphates in bioinorganic processes and the role of phosphoethanolamine and its complexes in the treatment of infections as well as cancer diseases.

## 2. Materials and Methods

### 2.1. Materials

O-phosphoethanolamine (enP) and cytidine (Cyd) from Sigma-Aldrich, uridine (Urd) from Merck and thymidine (Thd) from Fluka were used without further purification. Copper(II) nitrate from Merck was purified by recrystallization from water. The concentration of copper ions was determined by the method of Inductively Coupled Plasma Optical Emission Spectrometry (ICP OES). All solutions and experiments were prepared using demineralized, carbonate-free water.

### 2.2. Equilibrium Study

Potentiometric measurements were carried out using a Titrino 702 (Metrohm AG, Herisau, Switzerland) equipped with an autoburette with a combined Metrohm glass electrode. Prior to each series of measurements, the pH meter was calibrated with two standard buffer solutions of pH 4.002 and pH 9.225, and the electrode was calibrated in terms of H^+^ concentration [24]. All potentiometric titrations were made under helium (He 5.0) atmosphere at a constant ionic strength of µ = 0.1M (KNO_3_), a temperature of 20 ± 1 °C (the titration dish was placed in a thermostatic bath set at this temperature) and in the pH range from 2.5 to 11.0, using as a titrant CO_2_-free NaOH at a concentration of 0.2126 M. The concentration of copper(II) ions was 1 × 10^−3^ M, and the metal to ligand ratios were 2:1, 1:1, and 1:2 in the binary systems and 1:1:1 in the ternary systems. The protonation constants of the ligands and stability constants of the complexes were determined using the Hyperquad 2020 program, which uses the nonlinear method of least squares to minimize the sum (S) of the squares of the residuals between the observed quantities (*f*^obs^) and those calculated on the basis of the model (*f*^calc^),

$$S=\overset{n}{\underset{i=1}{\sum }}w_{i}{\left(f_{i}^{obs}-f_{i}^{calc}\right)}^{2}$$

where *n* is number of measurements and *w*_i_ is statistical weight [25].

The value of the determined ionic product of water was pK_w_ = 13.86. The stability constants of the complexes formed in binary and ternary systems could be evaluated by the following equilibria (charge was omitted for simplicity):

$$\mathrm{pL}+{\mathrm{qH}}^{+}\;↔L_{p}H_{q}\;\mathrm{pL}+qL\prime +{\mathrm{rH}}^{+}\;↔L_{p}{L\prime }_{q}H_{r}\;\beta =\frac{\left[L_{p}L_{q}^{\prime }H_{r}\right]}{{\left[L\right]}^{p}{\left[L\prime \right]}^{q}{\left[H\right]}^{r}}$$

All calculations were performed using 150–350 experimental points for each data set. The hydrolysis constant for copper(II) was taken from our previous publications [26,27]. For all systems, calculation began with the simplest hypothesis and was then extended by subsequent complex forms. After the improvement process, the set of complexes were established. The correctness of the model was confirmed by verification of the results described in the papers [28,29]. The distribution of particular forms was performed by the HYSS (Hyperquad Simulation and Speciation) program [30].

### 2.3. Visible (Vis) Spectroscopy

Samples for the visible studies were prepared in H_2_O at metal (M): ligand (L) ratio 1:1, 1:2 and 2:1 in binary systems and M:L:L′ = 1:1:1 in ternary systems (concentration of Cu(II) was 0.001–0.02 M). The spectra were recorded at room temperature in a PLASTIBRAND PMMA cell with 1 cm path length on an Evolution 300 UV–VIS ThermoFisher Scientific spectrometer equipped with a xenon lamp (range 450–950 nm, accuracy 0.2 nm, sweep rate 120 nm/min).

### 2.4. Electron Paramagnetic Resonance (EPR) Spectroscopy

EPR studies were carried out at −196 °C using glass capillary tubes (volume 130 µm^3^). The concentration of Cu(II) was 0.005M in water: glycol mixture (3:1) and the metal: ligand ratios were 1:1, 1:2 and 2:1 in binary and 1:1:1 in ternary systems. The spectra were recorded on an SE/X 2547 Radiopan instrument.

### 2.5. FT-IR Measurements

Samples for IR measurements were performed in D_2_O, and the pD of the solution was adjusted using NaOD or DCl, taking into account that pD = pH + 0.40 [31]. The metal concentration for the IR studies was 0.05 M and the ratio of M:L concentrations varied from 2:1, 1:1 and 1:2 in binary and 1:1:1 in ternary systems. The infrared spectra were taken on INVENIO R Bruker and collected in the range of 400–4000 cm^−1^ with ZnSe cells.

### 2.6. Raman Spectroscopy

Raman spectra measurements were performed in D_2_O with the pD adjusted similarly to the IR measurements. The metal and ligand concentration was 0.15M and the ratio of M:L and M:L:L′ was 1:1 and 1:1:1, respectively. The experiments were carried out using a Renishaw inVia Raman microscope equipped with an argon-ion laser, emitting a 785 nm wavelength, and a confocal DM 2500 Leica optical microscope. The Raman spectra were collected in the range of 200–3500 cm^−1^. The spectral resolution of the spectrometer was better than 2 cm^−1^. The positions of Raman peaks were calibrated before collecting the data using a Si sample as in internal standard. The exposure time of the CCD detector was 10 s.

### 2.7. NMR Measurements

Measurements of ^13^C and ^31^P NMR were performed in D_2_O and pD was adjusted similarly to the IR measurements. The concentration of the ligands was 0.1M, and the M:L molar ratio was 1:75 in binary systems and M:L:L′ 1:75:75 in ternary systems. NMR spectra were recorded on an Avance III Bruker 500 MHz spectrometer, using dioxane as an internal standard for ^13^C NMR and phosphoric acid for ^31^P NMR.

## 3. Results and Discussion

The structures of the studied ligands are presented in Table 1 and the values of successive protonation constants of phosphoethanolamine (enP) are presented in Table 2. Corresponding respectively to the protonation of -NH_2_ and -O-PO_3_^2−^, logK_1_ had a value of 10.41 and logK_2_ had a value of 5.70, which were determined by computer calculations from titration data and are consistent with the literature data [32,33,34].

Deprotonation of the first proton of -O-PO_3_H^−^ occurs at a pH value lower than was reached in this study and was not determined. The overall and successive protonation constants of phosphoethanolamine are given in Table 2.

The values for protonation and stability constants for complexes in the Cu(II)/Nuc systems were taken from our previous research and are given in Table 3 [35].

### 3.1. Cu(II)/enP Systems

The overall stability constants of the complexes formed in the studied systems were determined using the data obtained from the potentiometric titration (Table 4). The formation of M(HL) and ML(OH)_x_ type complexes was established. The computer calculation of the potentiometric titration data was performed taking into account the protonation constants of enP and the constants for Cu(II) hydrolysis (log*β* = −14.14 for Cu(OH)_2_) [17].

The coordination mode was established on the basis of the analysis of spectral parameters d-d transition energy in the UV–Vis spectra and g_‖_ as well as A_‖_ values in EPR studies (taking into consideration the relation of these values to the number of coordinated donor atoms). The conclusions were supported with the analysis of the IR spectra, changes in chemical shifts in the ^31^P and ^13^C NMR spectra of the ligand in complexes with respect to those of the free ligand, taking into regard our experience, and careful analysis of the results for paramagnetic ions [17,23,36].

**Figure 1:**
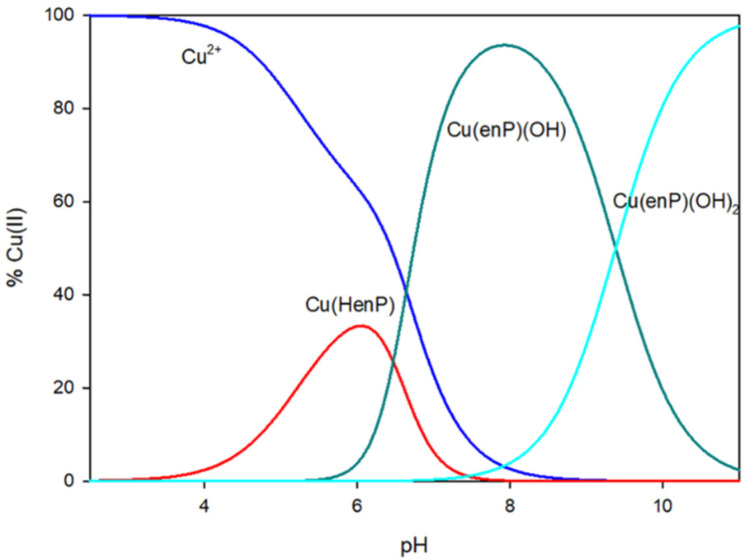
Distribution diagram for Cu(II) and phosphoethanolamine (ratio 1:1).

The protonated complex Cu(HenP) starts forming in the solution at pH close to 2.5. This form dominates at pH 6.0 and binds 35% of the copper ion in solution (Figure 1).

The Vis and EPR spectral parameters for the Cu(HenP) complex *(λ*_max_ = 799 nm, g_‖_ = 2.38 and A_‖_ = 143 × 10^−4^ cm^−1^, Table 5) indicate that only one oxygen atom is engaged in coordination. Analysis of changes in the chemical shifts in ^31^P and ^13^C (C(1) 0.28 ppm, C(2) −0.17 ppm and P −1.23 ppm) and in the IR spectrum confirmed that the phosphate group is involved in coordination (antisymmetric stretching band at 1086 cm^−1^ in the IR spectrum of the complex and at 1092 cm^−1^ in the spectrum of the free ligand [37]) (Table 6).

The NMR spectra also confirmed that the amine group is excluded from coordination at low pH (Table 5).

Cu(enP)(OH) started forming at pH 5.5 and dominated for 7.5–8.5, where it binded maximally to 95% of copper ions (Figure 1). The analysis of Vis spectral data (*λ*_max_ = 686 nm, Table 5) indicates {1N,1-2O} chromophore. Because of precipitation, it was not possible to take EPR spectra. A similar chromophore was detected in the copper(II) complexes with phosphoserine (Ser-P) and phosphothreonine (Thr-P) [23,27]. The involvement of phosphate and amine groups in the inner coordination sphere in the Cu(enP)(OH) complex was confirmed by NMR studies (C(1) −0.06 ppm, C(2) −0.14 ppm and P −0.7 ppm). The change in the mode of enP coordination was also noticeable in a significant increase in the logK_e_ value comparing to the Cu(HenP), which proves the participation of the nitrogen atom in the coordination. The complex stability increased: the logK_e_ value was 14.26 for Cu(enP)(OH) and the logK_e_ value was 2.88 for Cu(HenP). Copper has a greater affinity for nitrogen atoms than for oxygen atoms.

At pH 7.5, the next hydroxo complex started forming: Cu(enP)(OH)_2_. This complex dominated at pH close to 11.0, binding all copper ions introduced into the solution (Figure 1). The Vis spectra (*λ*_max_ = 661 nm) and analysis of changes in the chemical shifts in ^31^P and ^13^C (C(1) −2.72 ppm, C(2) 0.80 ppm and P −0.24 ppm) indicated that in the inner coordination sphere, besides nitrogen atom, are two oxygen atoms of the hydroxyl group (the phosphate group was excluded from the inner coordination sphere).

In summary, according to the spectral analysis, the main coordination site in the complexes, formed in the Cu(II)/enP system at low pH value, is the phosphate group. Increasing basicity leads to the exclusion of -O-PO_3_^2^^−^ of coordination. At higher pH values, the -NH_2_ becomes the main coordination site.

### 3.2. Complexes in the Ternary Systems

The stability constants of the complexes formed in the Cu(II)/enP/Nuc systems were calculated taking into account the protonation constants of the ligands and the overall stability constants (log*β*) of the complexes formed in the binary systems, Cu(II)/enP, Cu(II)/Urd, Cu(II)/Thd and Cu(II)/Cyd (Table 7) [35]. For pyrimidine nucleosides, the preferred site of protonation was the nitrogen atom N(3). The protonation constants of uridine and thymidine (logK_e_ = 9.22 and 9.79, respectively) were much higher than for cytidine (logK_e_ = 4.49). For enP, the potential coordination and non-covalent interaction centers were the same as in the binary systems—the phosphate and amine groups.

### 3.3. Cu(II)/enP/Urd System

**Figure 2:**
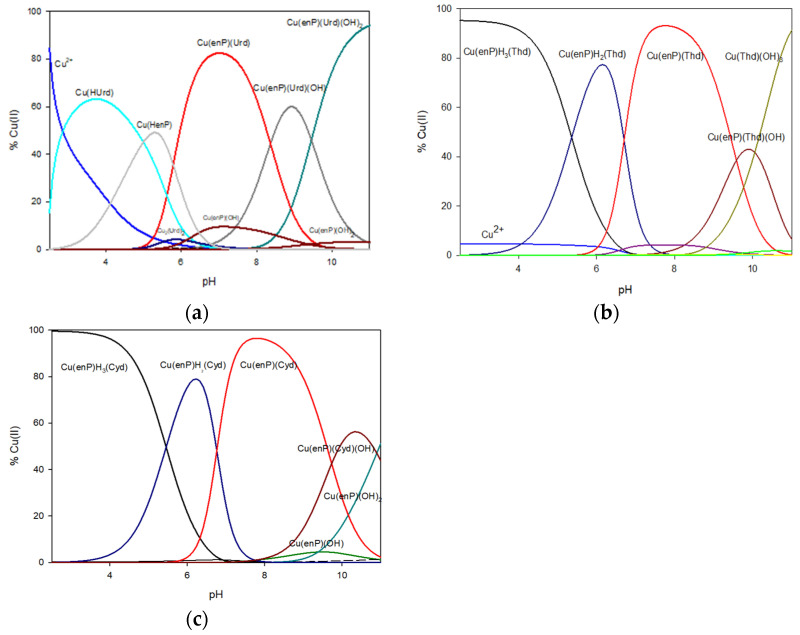
Distribution diagrams of the species formed in the systems: (**a**) Cu(II)/enP/Urd, (**b**) Cu(II)/enP/Thd and (**c**) Cu(II)/enP/Cyd. (For the sake of simplicity, ion charges in potentiometric description of the complexes were omitted.).

In the system containing copper(II) ions, phosphoethanolamine and uridine, three types of complexes were found: a simple complex MLL′ type and two hydroxo complexes MLL′(OH)_x_. The values of the overall stability constants are given in Table 7. In the lower pH value, only binary complexes Cu(HUrd) and Cu(HenP) were observing. The first ternary complex formed was Cu(II)(enP)(Urd) (logK_e_ = 12.74). It started forming at pH close to 5.0, accompanying deprotonation of ligands, and was dominant at the pH range between 6.0 to 8.0. At pH 7.0, this form bound more than 80% of the total copper ions introduced into the solution (Figure 2).

The results of the Vis and EPR studies (*λ*_max_ = 695 nm, g_‖_ = 2.37 and A_‖_ = 151 × 10^−4^ cm^−1^) indicate the formation of the species with {N, xO} chromophore (Table 8). The changes in the chemical shifts in the ^13^C NMR spectrum of the carbon atoms of uridine C(2) (−0.20 ppm) and C(4) (+0.07 ppm) point to the involvement of the N(3) nitrogen atom of uridine in coordination (Table 9).

This type of coordination was confirmed by IR spectra of the complex related to the spectra of the free ligand, where the positions of the IR stretching vibration bands assigned to the carbonyl groups (1655 cm^−1^ (ligand)/1653 cm^−1^ (complex) and 1693 cm^−1^ (ligand)/1699 cm^−1^ (complex) for C(2)=O and C(4)=O, respectively) shifted slightly. Moreover, the changes in the chemical shifts of the ^31^P signals of the phosphate group of enP (−2.28 ppm) indicate that copper was also coordinated to the phosphate group. The lack of significant changes in the Raman spectrum of Cu(II)/enP/Urd in the range characteristic for the amine group of enP (asymmetric stretching band at 983 cm^−1^ for the complex and 984 cm^−1^ for the free ligand) excludes the involvement of the nitrogen atom from enP [38].

The spectral parameters obtained for the Cu(II)(enP)(Urd)(OH) complex studies (*λ*_max_ = 668 nm, g_‖_ = 2.29 and A_‖_ = 178 × 10^−4^ cm^−1^) indicate {2N, xO} type coordination. This form of the complex was dominant at pH 9.0, binding 60% of the copper ions, and its stability constant logK_e_ was 5.46. The changes in chemical shifts in the ^13^C NMR spectrum (C(2) +0.25 ppm) of enP confirmed the participation of the amine group from enP in the coordination. Additionally, the shift in the ^31^P NMR decreases from −2.28 ppm in Cu(II)(enP)(Urd) to −0.09 ppm in the Cu(II)(enP)(Urd)(OH) complex indicates inactivity of the phosphate group. The participation of N(3) nitrogen atoms from uridine was confirmed by analysis of chemical shifts of ^13^C NMR (C(2) (−0.36 ppm), C(4) (+0.76 ppm)).

At pH 10.0, Cu(II)(enP)(Urd)(OH)_2_ started forming and became dominant at pH close to 11.0. The equilibrium constant of the Cu(II)(enP)(Urd)(OH)_2_ formation (logK_e_ = 4.35) was lower than that of the Cu(II)(enP)(Urd)(OH) (logK_e_ = 5.46). As follows from the position of *λ*_max_ = 668 nm, in MLL′(OH)_2_ complex types, coordination is the same as in MLL′(OH) type complexes, therefore {2N, xO} metal binds N(3) from Urd and nitrogen atoms from enP. The precipitate in higher concentrations makes it impossible to perform more studies and gather more information on the mode of coordination.

### 3.4. Cu(II)/enP/Thd System

**Figure 3:**
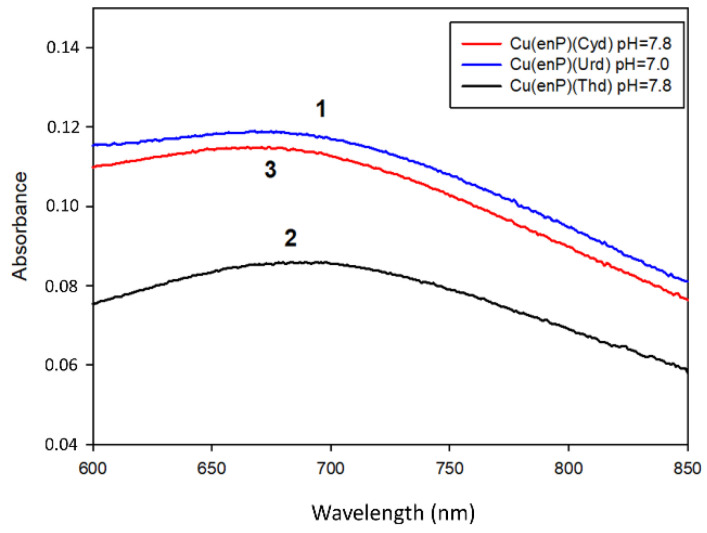
UV–Vis spectra of MLL′ forms of complexes in ternary systems.

**Figure 4:**
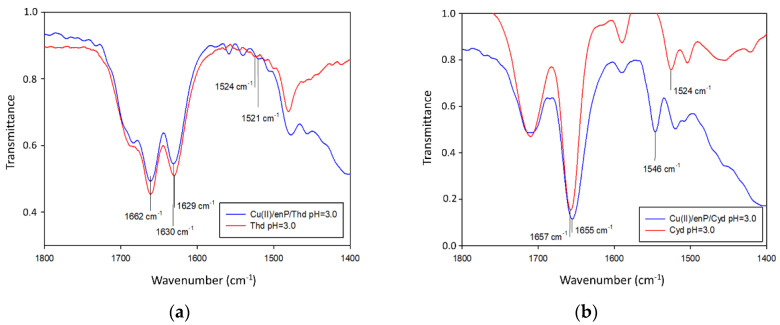
IR spectra of (**a**) Cu(enP)H_3_(Thd) and (**b**) Cu(enP)H_3_(Cyd) complexes compared to the free ligands.

In the Cu(II)/enP/Thd system, Cu(enP)H_3_(Thd), Cu(enP)H_2_(Thd), Cu(enP)(Thd) and Cu(enP)(Thd)(OH) complexes were found (stability constants are given in Table 7). The Cu(enP)H_3_(Thd) complex (logK_e_ = 9.97) occurred up to pH close to 7.0 and bound more than 90% of the total amount of copper(II) ions introduced at the beginning. According to the *λ*_max_ = 809 nm value and the EPR parameters of g_‖_ = 2.41 and A_‖_ = 147 × 10^−4^ cm^−1^ (Table 8, Figure 3), the inner coordination sphere comprised of only the oxygen atom of phosphate groups of enP (^31^P NMR shift −3.75 ppm), whereas the donor nitrogen atoms were not involved in coordination. The same mode of coordination as in the Cu(HenP) complex was observed as well as weak interactions between the protonated amine group of phosphoethanolamine (^13^C NMR C(2) −2.0 ppm) as the positive center and coordinated thymidine as the negative center. The weak interactions were confirmed by IR spectra where the changes in the positions of the *β* N(3)-H band (1524 cm^−1^ for the free ligand and 1521 cm^−1^ for the complex) were observed. No change in positions of the IR stretching vibration bands assigned to the carbonyl groups (1662 cm^−1^ for complex/1661 cm^−1^ for ligand and 1630–1629 cm^−1^ for both complex and ligand, for C(2)=O and C(4)=O, respectively) testifies to the lack of interactions of those groups of the bioligand with metal ions in the whole pH range considered [39] (Figure 4).

In the pH range from 3.0 to 8.0, the Cu(enP)H_2_(Thd) complex formed and at pH 6.0, bound 80% of Cu^2+^ in the solution. The EPR spectra parameters (g_‖_ = 2.38 and A_‖_ = 170 × 10^−4^ cm^−1^ and *λ*_max_ = 747 nm) indicated {2O} type chromophore, two of the donor oxygen atoms of the phosphate group being involved in the metalation (^31^P NMR shift −2.77 ppm). The changes in chemical shifts of ^13^C NMR in C(4) (+0.08 ppm) of thymidine and C(2) (−1.90 ppm) indicated that weak interactions between bioligands occurred. These interactions were confirmed by Raman spectra, where the band attributed to N-H and C-H in-plane bending was shifted from 1377 cm^−1^ in the free ligand to 1372 cm^−1^ in the complex, and the band attributed to C=O stretching and coupled to N-H and C-H asymmetric bending was shifted from 1661 cm^−1^ in the free ligand to 1665 cm^−1^ in the complex [40].

The Cu(enP)(Thd) complex was observed between the 6.0 and 11.0 pH range. It was dominant at pH close to 8.0 and bound almost all of the copper ions in the solution. The higher stability constant value for this complex formation (logK_e_ = 17.10), compared to the protonated species, points to a different mode of coordination. The spectral parameters of Cu(enP)(Thd) (*λ*_max_ = 680 nm and g_‖_ = 2.28) indicated the {2N, xO} chromophore (Figure 3).

Moreover, NMR analysis showed endocyclic N(3) atoms from thymidine (^13^C NMR of thymidine: C(2) +0.07 ppm, C(4) +0.18 ppm) and phosphate and amine groups from phosphoethanolamine (^13^C NMR: C(1) −1.96 ppm, C(2) −1.92 ppm, ^31^P NMR +3.23 ppm) are involved in coordination.

With increasing basicity of the solution, at pH = 8.0, the hydroxo complex Cu(enP)(Thd)(OH) started forming (logK_e_ = 4.27). An analysis of the results of the spectral studies (Vis *λ*_max_ = 674 nm and the EPR parameters of g_‖_ = 2.28 and A_‖_ = 175 × 10^−4^ cm^−1^) indicated that the inner coordination sphere is the same as in the Cu(enP)(Thd) complex {2N, xO}, with the addition of one oxygen atom of the hydroxyl group.

### 3.5. Cu(II)/enP/Cyd System

In the Cu(II)/enP/Cyd system, Cu(enP)H_3_(Cyd), Cu(enP)H_2_(Cyd), Cu(enP)(Cyd) and Cu(enP)(Cyd)(OH) complexes are formed (for stability constants see Table 7). The first protonated complex Cu(enP)H_3_(Cyd) (logK_e_ = 13.51) existed in the system from the beginning of the measurements and was dominant at pH close to 3.0, binding almost 100% of the copper ions. As follows from the d-d transition energy for this complex, *λ*_max_ = 798 nm and the EPR parameters (g_‖_ = 2.40, A_‖_ = 138 × 10^−4^ cm^−1^ (Table 8)), the metalation involved one oxygen atom. The changes in the chemical shift in the ^13^C NMR spectrum of phosphoethanolamine (C(1) 0.03 ppm, C(2) 0.61 ppm) and cytidine (C(2) 1.41 ppm, C(4) −0.62 ppm) as well as in the ^31^P NMR (−4.99 ppm) confirm the coordination of the copper(II) ion with a donor oxygen atom from the phosphate group of enP and weak interactions between endocyclic nitrogen atom N(3) from cytidine and the protonated amine group from enP. The IR spectra confirmed the weak interactions and shifts in the positions of the *β* N(3)-H band (1524 cm^−1^ for the free ligand and 1546 cm^−1^ for the complex) were observed. The positions of the IR stretching vibration bands assigned to the carbonyl groups (1655 cm^−1^ for the complex, 1657 cm^−1^ for the ligand) testify to the lack of interactions of these groups of the bioligand with metal ions in the whole pH range considered [39] (Figure 4).

By increasing pH value, the deprotonation of the cytidine N(3) atom occurs. The Cu(enP)H_2_(Cyd) complex started forming at pH 3.0 and became dominant at pH close to 6.2, binding almost the total amount of copper(II) ions introduced into the solution (Figure 2). Spectral parameters obtained from the Vis and EPR studies (*λ*_max_ = 746 nm, g_‖_ = 2.34, A_‖_ = 163 × 10^−4^ cm^−1^) indicated that in the inner coordination sphere, there are one nitrogen and one oxygen atom (Table 8). The changes in the chemical shift of the enP carbon atoms (C(1) −0.03 ppm, C(2) -0.08 ppm) and cytidine carbon atoms (C(2) −0.29 ppm, C(4) −0.10 ppm) indicate the participation of the nitrogen atom N(3) from cytidine and oxygen atom from the phosphate group of enP in coordination. The activity of the phosphate group was confirmed by a shift in ^31^P NMR spectrum (−7.35 ppm). The band assigned to the uncoordinated β N(3)-H group (1524 cm^−1^) underwent a significant shift in the spectra of the systems with copper ions (at 1520 cm^−1^), confirming the coordination of copper(II) to the N(3) atom.

The deprotonated complex Cu(enP)(Cyd), created from pH 6.0 and dominant at pH 7.8, bound more than 90% of metal ions in the solution (Figure 2). The value of *λ*_max_ decreased for this complex (*λ_max_* = 665 nm) compared to the Cu(enP)H_2_(Cyd) form (*λ*_max_ = 746 nm) and with the EPR spectral parameter change (g_‖_ = 2.32, A_‖_ = 160 × 10^−4^ cm^−1^), this points to the participation of the two nitrogen atoms in the inner coordination sphere. The chemical shift in the ^31^P NMR decrease from −7.35 ppm to −0.95 ppm and shifts in the ^13^C NMR at the enP carbon atoms (C(1) −0.08 ppm, C(2) −0.07 ppm) mean that the nitrogen atom from phosphoethanolamine became more effective in metalation than phosphate. According to the chemical shifts in the ^13^C NMR (C(2) 0.02 ppm, C(4) −0.09 ppm for Cyd), the N(3) atoms of cytidine also took part in the complexation of the copper ions.

With increasing basicity of the solution, at pH = 8.0, the hydroxo complex Cu(enP)(Cyd)(OH) starts forming (logK_e_ = 4.12). This complex bound more than 50% of Cu(II) at pH 10.3. The results of the spectral studies and NMR changes (*λ*_max_ = 645 nm, ^13^C NMR for enP: C(1) −0.9 ppm, C(2) −1.16 ppm) indicate that the inner coordination sphere is the same as in Cu(enP)(Thd) {2N, xO} in that one oxygen atom from the hydroxyl group is additionally involved. A significant reduction in the value of the shift in the spectrum ^31^P NMR from −0.95 ppm to −0.03 ppm point to an even greater drop in activity of the phosphate group in complexation.

## 4. Conclusions

The formation of binary complexes of phosphoethanolamine and copper(II) ions has been established. The MHL, ML as well as M_2_L_2_ and ML(OH)_x_ complex types were confirmed in the studied systems. In the complexes formed at low value pH, the phosphate group was involved in the metalation. At the physiological pH value, a different type of coordination was observed. This process was a result of changes in the effectiveness of the competing donor groups. The carboxyl and amine groups of the ligands were involved in the coordination, while the activity of the phosphate group considerably decreased.

In the ternary systems (Cu/Nuc/enP), the mode of complexation directly depended on pH. Below the physiological pH, phosphoethanolamine coordinated only via the phosphate group and with increasing basicity, the main reaction center becomes the amine group.

It is noteworthy that the ternary complex with uridine showed a lower value of the stability constant than the analogous forms with cytidine and thymidine. Interestingly, in the case of the complex with uridine, only one nitrogen atom was involved in the internal coordination sphere and enP was coordinated via a phosphate group. Moreover, in the system with Cyd and Thd at low pH, the formation of molecular complexes was established.

## Figures and Tables

**Table 1:** The structures of phosphoethanolamine, uridine, thymidine and cytidine.

Compound Name	Abbreviation	Structural Formula
2-Aminoethyl dihydrogen phosphate(Phosphoethanolamine)	enP	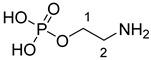
1-[(2*R*,3*R*,4*S*,5*R*)-3,4-dihydroxy-5-(hydroxymethyl)oxolan-2-yl]pyrimidine-2,4-dione (Uridine)	Urd	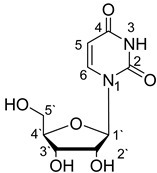
1-[(2R,4S,5R)-4-Hydroxy-5-(hydroxymethyl)oxolan-2-yl]-5-methylpyrimidine-2,4(1H,3H)-dione(Thymidine)	Thd	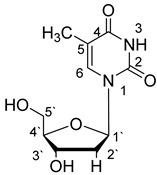
4-Amino-1-[(2R,3R,4S,5R)-3,4-dihydroxy-5-(hydroxymethyl)oxolan-2-yl]pyrimidin-2(1H)-one (Cytidine)	Cyd	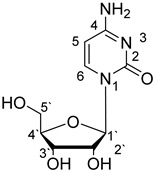

**Table 2:** Overall and successive protonation constants of enP (standard deviation is given in parenthesis).

Species	Overall Protonation Constants (log*β*)	Reactions	Successive Protonation Constants logK_e_ logK_e_*	
H(enP)	10.41 (2)	enP + H^+^ ⇄ H(enP)	10.41	10.12 [32], 10.26 [34]
H_2_(enP)	16.11 (3)	H(enP) + H^+^ ⇄ H_2_(enP)	5.70	5.52 [32], 5.77 [34]

logK_e_* from literature data [32,34].

**Table 3:** Protonation and stability constants for complexes in the Cu(II)/Nuc systems (Standard deviation is given in parenthesis) [35].

Species	Urd	Thd	Cyd
H_2_L	-	-	-
HL	9.22	9.79	4.49
MHL	11.55	-	-
ML	4.32	5.67	2.25
M_2_L_2_	12.86	-	-
ML(OH)	−3.72	−1.92	-
ML(OH)_2_	−12.96	−9.76	−12.09
ML(OH)_3_	−23.78	−16.48	-

**Table 4:** Protonation constants of enP, overall and successive stability and equilibrium constants of Cu(II) complexes (standard deviation is given in parenthesis).

Species	Overall Stability Constants log*β*	Reactions	logK_e_	logK_e_*
Cu(HenP)	13.29 (7)	Cu^2+^ + (HenP) ⇆ Cu(HenP)	2.88	2.54 [32]
Cu(enP)(OH)	0.40 (2)	Cu^2+^ + enP + H_2_O ⇆ Cu(enP)(OH) + H^+^	14.26	N.D.
Cu(enP)(OH)_2_	−7.26 (4)	Cu(enP)(OH) + H_2_O ⇆ Cu(enP)(OH)_2_ +H^+^	6.20	N.D.

**Table 5:** Spectral parameters for binary system Cu(II)/enP.

Species	pH	*λ*_max_ [nm]	ε [dm^3^ mol^−1^ cm^−1^]	g_‖_	A_‖_ [10^−4^ cm^−1^]	Chromophore
Cu(HenP)	6.0	799	22	2.38	143	{1-2O}
Cu(enP)(OH)	8.0	686	82	-	-	{1N,1-2O}
Cu(enP)(OH)_2_	10.5	661	78	-	-	{1N, 2O}

**Table 6:** NMR differences between signal positions for the ligand in the complex in relation to the free ligand (ppm).

Species	pH	C(1)	C(2)	P
Cu(HenP)	6.0	0.28	−0.17	−1.23
Cu(enP)(OH)	8.0	−0.06	−0.14	−0.70
Cu(enP)(OH)_2_	10.5	−2.72	0.80	−0.24

**Table 7:** Overall and successive stability as well as equilibrium constants of Cu(II) complexes in the Cu(II)/enP/Nuc systems (standard deviation is given in parenthesis).

Species	Overall Stability Constants log*β*	Reactions	logK_e_
Cu(enP)(Urd)	12.74 (6)	Cu^2+^ + (enP) + (Urd) ⇆ Cu(enP)(Urd)	12.74
Cu(enP)(Urd)(OH)	4.34 (6)	Cu(enP)(Urd) + H_2_O ⇆ Cu(enP)(Urd)(OH) + H^+^	5.46
Cu(enP)(Urd)(OH)_2_	−5.17 (5)	Cu(enP)(Urd)(OH) + H_2_O ⇆ Cu(enP)(Urd)(OH)_2_ + H^+^	4.35
Cu(enP)H_3_(Thd)	35.87 (2)	Cu^2+^ + (H_2_enP) + (HThd) ⇆ Cu(enP)H_3_(Thd)	9.97
Cu(enP)H_2_(Thd)	30.51 (1)	Cu(HenP) + (HThd) ⇆ Cu(enP)H_2_(Thd)	7.43
Cu(enP)(Thd)	17.10 (1)	Cu^2+^ + (enP) + (Thd) ⇆ Cu(enP)(Thd)	17.10
Cu(enP)(Thd)(OH)	7.51 (1)	Cu(enP)(Thd) + H_2_O ⇆ Cu(enP)(Thd)(OH) + H^+^	4.27
Cu(enP)H_3_(Cyd)	34.11 (4)	Cu^2+^ + (H_2_enP) + (HCyd) ⇆ Cu(enP)H_3_(Cyd)	13.51
Cu(enP)H_2_(Cyd)	28.66 (3)	Cu(HenP) + (HCyd) ⇆ Cu(enP)H_2_(Cyd)	10.88
Cu(enP)(Cyd)	15.13 (3)	Cu^2+^ + (enP) + (Cyd) ⇆ Cu(enP)(Cyd)	15.13
Cu(enP)(Cyd)(OH)	5.39 (4)	Cu(enP)(Cyd) + H_2_O ⇆ Cu(enP)(Cyd)(OH) + H^+^	4.12

**Table 8:** Spectral parameters for complexes formed in the ternary Cu(II)/enP/Nuc systems.

Species	pH	*λ*_max_ [nm]	ε [dm^3^ mol^−1^ cm^−1^]	g_‖_	A_‖_ [10^−4^ cm^−1^]	Chromophore
Cu(enP)(Urd)	7.0	695	59.41	2.37	151	{1N, xO}
Cu(enP)(Urd)(OH)	9.0	668	79.35	2.29	178	{2N, xO}
Cu(enP)(Urd)(OH)_2_	11.0	638	89.07	-	-	{2N, xO}
Cu(enP)H_3_(Thd)	3.0	809	12.00	2.41	147	{1O}
Cu(enP)H_2_(Thd)	6.2	747	22.00	2.38	170	{2O}
Cu(enP)(Thd)	7.8	680	57.00	2.28	-	{2N, xO}
Cu(enP)(Thd)(OH)	10.3	674	62.00	2.28	175	{2N, xO}
Cu(enP)H_3_(Cyd)	3.0	798	12.46	2.40	138	{1O}
Cu(enP)H_2_(Cyd)	6.2	746	21.18	2.34	163	{1N, 1O}
Cu(enP)(Cyd)	7.8	665	76.66	2.32	160	{2N, xO}
Cu(enP)(Cyd)(OH)	10.3	645	94.67	-	-	{2N, xO}

**Table 9:** NMR differences between signal positions for the ligand in the complex in relation to the free ligand (ppm).

					enP			Nucleoside									
System				pH	C1	C2	P	C2	C4	C5	C6	C1′	C2′	C3′	C4′	C5′	CH_3_
**Cu(II)/enP/Urd**				**7.0**	+0.02	−0.05	−2.28	−0.2	+0.07	−0.25	+0.01	−0.11	+0.01	0.00	−0.03	−0.01	
				**9.0**	0.00	0.25	−0.09	−0.36	+0.73	−0.07	+0.02	−0.38	−0.03	−0.03	−0.05	−1.40	
**Cu(II)/enP/Cyd**				**3.0**	0.03	0.61	−4.99	−1.41	−0.62	−	0.12	−0.94	−0.07	−0.04	0.05	−0.01	
				**6.2**	−0.03	−0.08	−7.35	−0.29	−0.10	−	0.00	−0.27	0.00	0.00	−0.03	0.01	
				**7.5**	−0.08	−0.07	−0.95	0.02	−0.09	−0.24	0.01	0.02	0.00	−0.01	−0.02	−0.02	
				**10.5**	−0.90	−1.16	−0.03	0.02	0.00	−0.2	0.00	0.05	0.00	−0.01	−0.03	−0.02	
**Cu(II)/enP/Thd**				**3.0**	−1.94	−2.00	−3.75	−0.01	0.01	−0.01	0.00	0.00	0.01	0.00	0.01	−0.01	0.00
				**6.2**	−1.85	−1.90	−2.77	0.00	0.08	−0.02	0.00	−0.03	−0.03	0.00	−0.03	−0.01	−0.05
				**7.8**	−1.96	−1.92	3.23	0.07	0.18	−0.02	0.01	−0.02	0.01	0.02	−0.01	0.01	−0.01

## Data Availability

Not applicable.
